# Supplementary material for: Numerical simulation of rock blasting under different in-situ stresses and joint conditions
Source: PLoS One. 2024 Apr 22;19(4):e0299258. doi: 10.1371/journal.pone.0299258 (PMC11034639; doi:10.1371/journal.pone.0299258)
Supplement: Attachment — Submitted filename: Plos review paper.pdf [file pone.0299258.s001.pdf]

PLOS ONE

Numerical simulation of rock blasting under different ground stresses and open joint structures

--Manuscript Draft--

**Manuscript Number:**	PONE-D-23-39104
**Article Type:**	Research Article
**Full Title:**	Numerical simulation of rock blasting under different ground stresses and open joint structures
**Short Title:**	Opening joints and rock blasting
**Corresponding Author:**	Nannan Li<br>Liaoning University of Engineering and Technology: Liaoning Technical University<br>Fuxin, Liaoning Province CHINA
**Keywords:**	air joint; Joint thickness; Initial in-situ stress; Joint Angle; Numerical Simulation; Crack propagation
**Abstract:**	There are a large number of naturally formed joints in rock mass, a numerical model of jointed rock mass was established using LS-DYNA software to investigate the evolution of blasting damage under various in-situ stresses and open joints. The study considered different joint thicknesses (2cm, 4cm, and 6cm), joint angles (0°, 30°, 60°, and 90°), and in-situ stress conditions (lateral stress coefficients of 0.5, 1, and 2, with vertical in-situ stresses of 10MPa and 20MPa). The research aimed to understand the mechanisms underlying crack initiation and propagation. The results show that: (1) Joint thickness influences the expansion of blasting cracks. Thinner joints result in intersecting joint planes under pressure, with maximum stress occurring at the center of the gun hole connection. In contrast, thicker joints lead to concentrated stress at the joint ends and even stress distribution within the rock mass. (2) When there is no joint, the initial stress field has a certain guiding effect on the crack growth, and the crack growth tends to the maximum in-situ stresses direction; (3) The presence of joints exerts a barrier effect on the expansion and penetration of cracks. When explosion stress waves reach the joint surface, their propagation is impeded, leading to the diffusion of wing cracks at the joint ends. When the lateral stress coefficient and joint angle are the same, an increase in initial in-situ stress results in a reduction in the area of the blasting damage zone. (4) Under the same initial in-situ stress conditions, the area of the blasting damage zone initially increases and then decreases with an increasing joint angle. However, it remains larger than that without a joint, and there exists an optimal angle that maximizes the damage area. In the simulated conditions, the area of damage cracks is greatest when the joint angle is 60° dip angle. (5) The presence of initial in-situ stress has a certain impact on the initiation and expansion of blasting cracks. The degree and nature of this influence are not solely related to the lateral stress coefficient but also depend on the joint's angle and thickness. When in-situ stress is present, the initial in-situ stress field's pressure is not conducive to the initiation and propagation of blasting cracks. However, the existence of a joint has a noticeable guiding and promoting effect on crack propagation, and the pattern of crack propagation is influenced by both joint and in-situ stress conditions.
**Order of Authors:**	Hai Rong
	Nannan Li
	Chen Cao
	Yadi Wang
	Jincheng Li
	Mingda Li
**Additional Information:**	
**Question**	**Response**
**Financial Disclosure**	The author(s) received no specific funding for this work.

Powered by Editorial Manager® and ProduXion Manager® from Aries Systems Corporation

Enter a financial disclosure statement that describes the sources of funding for the work included in this submission. Review the submission guidelines for detailed requirements. View published research articles from *PLOS ONE* for specific examples.<br><br>This statement is required for submission and **will appear in the published article** if the submission is accepted. Please make sure it is accurate.<br><br>**Funded studies**<br>Enter a statement with the following details:<br>• Initials of the authors who received each award<br>• Grant numbers awarded to each author<br>• The full name of each funder<br>• URL of each funder website<br>• Did the sponsors or funders play any role in the study design, data collection and analysis, decision to publish, or preparation of the manuscript?<br><br>Did you receive funding for this work?	
**Competing Interests**<br><br>Use the instructions below to enter a competing interest statement for this submission. On behalf of all authors, disclose any competing interests that could be perceived to bias this work—acknowledging all financial support and any other relevant financial or non-financial competing interests.<br><br>This statement is required for submission and **will appear in the published article** if the submission is accepted. Please make sure it is accurate and that any funding sources listed in your Funding Information later in the submission form are also declared in your Financial Disclosure statement.	The authors have declared that no competing interests exist.

View published research articles from *PLOS ONE* for specific examples.<br><br>**NO authors have competing interests**<br><br>Enter: *The authors have declared that no competing interests exist.*<br><br>**Authors with competing interests**<br><br>Enter competing interest details beginning with this statement:<br><br>*I have read the journal's policy and the authors of this manuscript have the following competing interests: [insert competing interests here]*<br><br>* typeset	
**Ethics Statement**<br><br>Enter an ethics statement for this submission. This statement is required if the study involved:<br><br>• Human participants<br>• Human specimens or tissue<br>• Vertebrate animals or cephalopods<br>• Vertebrate embryos or tissues<br>• Field research<br><br>Write "N/A" if the submission does not require an ethics statement.<br><br>General guidance is provided below. Consult the submission guidelines for detailed instructions. **Make sure that all information entered here is included in the Methods section of the manuscript.**	"N/A"

**Format for specific study types**<br><br>**Human Subject Research (involving human participants and/or tissue)**<br>• Give the name of the institutional review board or ethics committee that approved the study<br>• Include the approval number and/or a statement indicating approval of this research<br>• Indicate the form of consent obtained (written/oral) or the reason that consent was not obtained (e.g. the data were analyzed anonymously)<br><br>**Animal Research (involving vertebrate animals, embryos or tissues)**<br>• Provide the name of the Institutional Animal Care and Use Committee (IACUC) or other relevant ethics board that reviewed the study protocol, and indicate whether they approved this research or granted a formal waiver of ethical approval<br>• Include an approval number if one was obtained<br>• If the study involved *non-human primates*, add *additional details* about animal welfare and steps taken to ameliorate suffering<br>• If anesthesia, euthanasia, or any kind of animal sacrifice is part of the study, include briefly which substances and/or methods were applied<br><br>**Field Research**<br><br>Include the following details if this study involves the collection of plant, animal, or other materials from a natural setting:<br>• Field permit number<br>• Name of the institution or relevant body that granted permission	
**Data Availability**<br><br>Authors are required to make all data underlying the findings described fully available, without restriction, and from the time of publication. PLOS allows rare exceptions to address legal and ethical concerns. See the PLOS Data Policy and FAQ for detailed information.	Yes - all data are fully available without restriction

A Data Availability Statement describing where the data can be found is required at submission. Your answers to this question constitute the Data Availability Statement and **will be published in the article**, if accepted.<br><br>**Important:** Stating 'data available on request from the author' is not sufficient. If your data are only available upon request, select 'No' for the first question and explain your exceptional situation in the text box.<br><br>Do the authors confirm that all data underlying the findings described in their manuscript are fully available without restriction?	
**Describe where the data may be found in full sentences. If you are copying our sample text, replace any instances of XXX with the appropriate details.**<br><br>• If the data are **held or will be held in a public repository**, include URLs, accession numbers or DOIs. If this information will only be available after acceptance, indicate this by ticking the box below. For example: *All XXX files are available from the XXX database (accession number(s) XXX, XXX.).*<br>• If the data are all contained **within the manuscript and/or Supporting Information files**, enter the following: *All relevant data are within the manuscript and its Supporting Information files.*<br>• If neither of these applies but you are able to provide **details of access elsewhere**, with or without limitations, please do so. For example:<br><br>*Data cannot be shared publicly because of [XXX]. Data are available from the XXX Institutional Data Access / Ethics Committee (contact via XXX) for researchers who meet the criteria for access to confidential data.*<br><br>*The data underlying the results presented in the study are available from (include the name of the third party*	All relevant data are within the manuscript and its Supporting Information files.

and contact information or URL).<br>• This text is appropriate if the data are owned by a third party and authors do not have permission to share the data.<br><br>* typeset	
Additional data availability information:	

Powered by Editorial Manager® and ProduXion Manager® from Aries Systems Corporation

Manuscript Click here to access/download;Manuscript;Manuscript.docx

Numerical simulation of rock blasting under different in-situ stresses and joint conditions

Hai Rong[1], Nannan Li[1], Chen Cao[1], Yadi Wang[1] & Jincheng Li[1], Mingda Li[1]

(1. *College of Mining*, *Liaoning Technical University*, *Fuxin* 123000, *China*)

Correspondence should be addressed to Hai Rong: ronghai1988@163.com

Abstract: There are a large number of naturally formed joints in rock mass, a numerical model of jointed rock mass was established using LS-DYNA software to investigate the evolution of blasting damage under various in-situ stresses and open joints. The study considered different joint thicknesses (2cm, 4cm, and 6cm), joint angles (0°, 30°, 60°, and 90°), and in-situ stress conditions (lateral stress coefficients of 0.5, 1, and 2, with vertical in-situ stresses of 10MPa and 20MPa). The research aimed to understand the mechanisms underlying crack initiation and propagation. The results show that: (1) Joint thickness influences the expansion of blasting cracks. Thinner joints result in intersecting joint planes under pressure, with maximum stress occurring at the center of the gun hole connection. In contrast, thicker joints lead to concentrated stress at the joint ends and even stress distribution within the rock mass. (2) When there is no joint, the initial stress field has a certain guiding effect on the crack growth, and the crack growth tends to the maximum in-situ stresses direction; (3) The presence of joints exerts a barrier effect on the expansion and penetration of cracks. When explosion stress waves reach the joint surface, their propagation is impeded, leading to the diffusion of wing cracks at the joint ends. When the lateral stress coefficient and joint angle are the same, an increase in initial in-situ stress results in a reduction in the area of the blasting damage zone. (4) Under the same initial in-situ stress conditions, the area of the blasting damage zone initially increases and then decreases with an increasing joint angle. However, it remains larger than that without a joint, and there exists an optimal angle that maximizes the damage area. In the simulated conditions, the area of damage cracks is greatest when the joint angle is 60° dip angle. (5) The presence of initial in-situ stress has a certain impact on the initiation and expansion of blasting cracks. The degree and nature of this influence are not solely related to the lateral stress coefficient but also depend on the joint's angle and thickness. When in-situ stress is present, the initial in-situ stress field's pressure is not conducive to the initiation and propagation of blasting cracks. However, the existence of a joint has a noticeable guiding and promoting effect on crack propagation, and the pattern of crack propagation is influenced by both joint and in-situ stress conditions.

usually in abstract include methodology before results

the results finding is good. However double check the max wording in the abstract

Key words: air joint; Joint thickness; Initial in-situ stress; Joint Angle; Numerical simulation; Crack propagation

0 Introduction

Mesomorphic rock mass is widely distributed in nature, accounting for about 66.7% of the land area, and 77.3% in China [1]. The horizontal stratified rock mass has the characteristics of transverse isotropy, and there are structural weak planes inside the rock mass. The composition of the rock mass is basically the same parallel to the structural plane, while the direction perpendicular to the structural plane shows frequent alternations of soft and hard. At the same time, there are many joints and cracks in natural rock mass, resulting in obvious anisotropy in the aspects of force and deformation [2-5].

Currently, blasting remains the primary method for rock fragmentation. In the process of rock breaking, as the propagation distance increases, the shock wave generated by explosive detonation rapidly transforms into a stress wave [6], playing a crucial role in the rock fragmentation process. The presence of naturally occurring joints, cracks, bedding, faults, and other structural features within natural rock masses results in variations in the mechanical properties, vibrations, permeability, and energy transfer characteristics of these rock formations [7-9]. The propagation and attenuation of explosion-induced stress waves within such rock masses, which contain joints, cracks, and faults, are also subject to alteration due to these structural features, ultimately impacting the effectiveness and safety of engineering blasting operations [10-12]. Therefore, it is of great significance to study the law of explosive crack propagation and stress wave propagation of jointed rock mass under explosion load to improve the blasting energy utilization efficiency, rock breaking effect and safety of rock mass engineering [13]. Domestic and foreign scholars have conducted in-depth studies on this. Chai Shaobo et al [14] compared the propagation theory of explosion stress waves in rock mass with cross-jointed joints with numerical models, and further discussed the propagation law of explosion stress waves in rock mass with cross-jointed joints. Wang Shumin et al [15] introduced the Poyting-Thomson model as the discontinuity condition and derived the propagation equation of stress waves through a set of parallel viscoelastic joints based on the time-domain recursion method to explore the influence of viscoelastic joints on the propagation of stress waves in rock mass. Xu Bangshu et al [16] studied the blasting parameters of horizontal layered rock mass with large section where joint fissured development, carried out field blasting tests and the failure mechanism analysis of layered rock mass, optimized the smooth blasting parameters of tunnel excavation, cut hole layout scheme and maximum single-hole charge parameter, the characteristics of surrounding rock, overcut and undercut of tunnel contour and deformation of surrounding rock after explosion are compared and analyzed. Niktabar et al [17] used a large direct shear testing machine to study the shear properties of jointed rock mass in different roughness seasons, aiming at the fact that the jointed rock mass is often subjected to dynamic loading by blasting during mining. The results show that the shear strength of regular joints is higher than that of ordinary joints, and the shear strength of rock mass decreases with the increase of shear times. Roy et al [18] conducted experimental studies on fracture failure modes of jointed rock masses with different fracture toughness and tensile strength, and the results showed that: With the decrease of joint thickness, the fracture toughness and tensile strength of rock mass decrease, and the fracture process zone of jointed rock mass is less sensitive to the direction of the joint, which is related to the thickness of the joint. The interaction of uneven surfaces at the joint increases the friction resistance and dissipates the fracture energy of the sample. Yang et al [19] studied the blasting crack propagation characteristics of jointed rock mass under high stress conditions by using a digital laser

dynamic caustic line experiment system, obtained the crack initiation mode, stress intensity factor and crack velocity at the joint, and concluded that the state of high stress significantly increased the shear failure degree of crack initiation at the joint, resulting in an increase in the crack initiation Angle. Miranda et al [20] analyzed the influence of joint geometry and number on acoustic wave propagation through acoustic wave test. The test results of Singh and Sastry [21] show that the average block degree of blasting is controlled by the intersection Angle between the structural plane and the free plane. In the range of 0° to 90°, the average block degree increases with the increase of the intersection Angle.

Some scholars have studied the influence of joint on explosive crack propagation and stress wave propagation in rock mass by numerical simulation. For example, Xie Bing et al [22] and Qu Shijie et al [23] respectively simulated and analyzed the influence of joint geometric parameters on the pre-cracking joint formation effect, and concluded that the greater the Angle of joint group and gun hole connection, the better the joint formation effect. Under the same conditions, the smaller the joint Angle and spacing, the more difficult it is to form connected cracks, and the easier it is to form sawtooth cracks. Wei Chenhui et al [24] and Zhang Fengpeng et al [25] respectively studied the propagation of blasting cracks in rock mass under different initial stresses and joint characteristic parameters, and the results showed that the reflection tensile failure of the joint surface was weaker than that without filling. The coupling between the tensile crack generated by the reflection tensile action of the joint surface and the blasting main crack enhanced the fracture degree of the rock mass between the explosion source and the joint. The existence of the joint is conducive to the propagation of the explosive crack along the joint plane. Deng et al [26] used DEM method to conduct numerical simulation on the damage of circular tunnel under the action of explosion shock wave, and studied the spatial mechanical characteristics of jointed rock mass, the initial stress of rock mass and the impact of shock wave amplitude on tunnel damage. The results show that the joint direction in the rock mass surrounding the tunnel has a great influence on tunnel damage. The initial stress of the tunnel has little influence on the damage of the tunnel, and the bolting support can greatly improve the stability of the tunnel.

In summary, existing research in the field of the blasting mechanism of jointed rock masses has primarily focused on experimental investigations. While there have been extensive discussions in numerical simulations regarding joint parameters such as thickness, angle, numerical control, filling strength, and length, a noticeable gap remains in the study of joint parameters specifically for double-hole single joints under various conditions. Building upon the work of previous researchers, this paper aims to further explore the patterns of crack propagation and the propagation of explosive stress waves during the blasting process of jointed rock masses. Additionally, it takes into account different in-situ stress conditions and conducts numerical simulations to study the influence of in-situ stress conditions, joint thickness, and joint angle (the angle between the joint plane and the hole axis) on the initiation and propagation of explosive cracks.

1. Establishment of numerical model

1.1 Calculation model

In this study, a computational model incorporating joint surfaces was established using the finite element software LS-DYNA. The specific model is illustrated in Figure 1, with dimensions of 700cm×600cm. Two circular holes with a diameter of 100cm were excavated within the model, and the spacing between the holes was set at 300cm, and the open (empty) joint is set between the two holes (the distance between the holes is adjusted to 500 cm to keep the joint length unchanged

considering that the Angle of the joint is 0°). Non-reflective boundary conditions were applied to the perimeter of the model, with dimensions given in g-cm-us units. To better illustrate the impact of the joint on explosive stress waves, monitoring points were strategically positioned within the computational model. Monitoring points were selected at the joint surface, with one monitoring point designated for every 10 units along the joint. The model employed SOLID 164 hexahedron units, the ALE algorithm was utilized for modeling explosives and air, and a coupled analysis was conducted between explosives, air, and the rock mass.

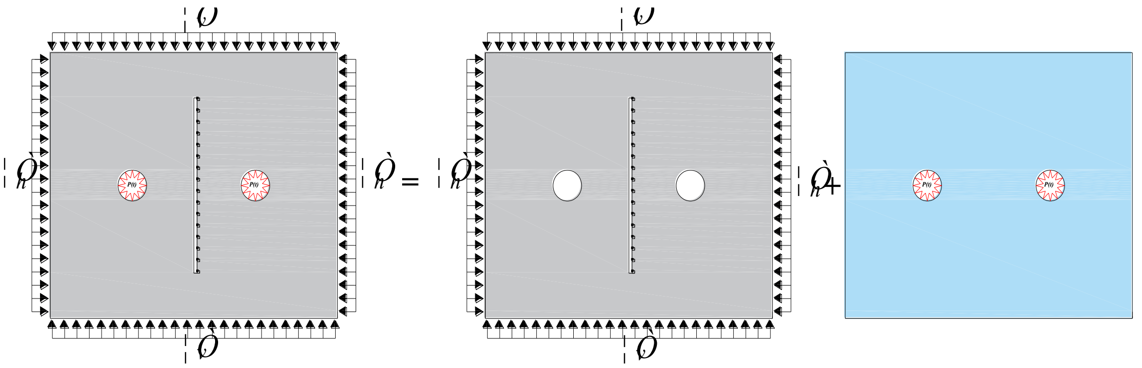

* improve Fig 1
'is it 6v } 6H ?

Fig 1. Numerical model

1.2 Explosive and air parameters and equation of state

The high performance explosive material model *MAT_HIGH_EXPLOSIVE_BURN bu ilt in ANSYS/LS-DYNA software and the JWL equation of state are used to describe the volume, pressure and energy characteristics of the explosive products during the explosion. The expression is as follows:

$$P = A\left(1-\frac{\omega}{R_1V}\right)\exp(-R_1V)+B\left(1-\frac{\omega}{R_2V}\right)\exp(-R_2V)+\frac{\omega E_0}{V}$$

Where: P is the detonation pressure; V is the relative volume; E_0 is the initial specific internal energy; A, B, R_1, R_2, ω are material constants, as shown in Table 1.

Table 1 The parameters of explosive

[illegible]$_e$/(kg/m^3)	V_0D(m/s)	P_{CJ}/GPa	A/GPa	B/GPa	R1	R2	ω	E_0/GPa
1320	6690	16	586	21.6	5.81	1.77	0.282	7.38

Air adopts the empty matter material model *MAT_NULL, and its equation of state is expressed in linear polynomial *EOS_LINER_POLYNOMIAL:

$$P = C_0 + C_1V + C_2V^2 + C_3V^3 + \left(C_4 + C_5V + C_6V^2\right)E_0$$

In the formula, C_0~C_6 are the relevant parameters of the equation. Among them, C_4=C_5=0.4, E_0=2500MJ/m3, V=1.0, and other parameters are 0.

1.3 Rock Parameters

what type of Mesormophic rock is simulated?

The geometric model involves three distinct materials: rock, explosives, and air. Rock materials are characterized as porous and brittle, featuring numerous pores and micro-cracks. When subjected to external forces, these cracks undergo progressive development and interpenetration, culminating in the formation of extensive macroscopic fractures, which ultimately lead to material damage and

failure. The fracture development process in rock materials consists of several distinct stages, including elasticity mechanics, fracture mechanics, and damage mechanics [27]. RHT model is a tensile and compressive damage model proposed by Riedel, Hiermaier and Thoma et al [28] based on the HJC model, which considers the impact of the failure strength of rock materials under blasting and dynamic loads on the impact pressure, strain rate, strain hardening and damage softening of rock materials. The study of WANG et al [29] shows that RHT model has good applicability to the blasting simulation of rock materials. In this paper, RHT model is used for numerical simulation research, and the specific parameters [30] are shown in Table 2.

Table 2 RHT constitutive model parameters for rock [30]

Parameter	Value	Parameter	Value
Mass density (kg/m^3)	2660	Break compressive strain rate	3E+25
Elastic shear modulus (GPa)	21.9	Break tensile strain rate	3E+25
Relative shear strength	0.18	Lode angle dependence factor Q0	0.68
Relative tensile strength	0.04	Lode angle dependence factor B	0.01
Parameter for polynomial EOS T1 (GPa)	35.27	Compressive yield surface parameter	0.53
Parameter for polynomial EOS T2 (GPa)	0	Tensile yield surface parameter	0.7
Damage parameter D1	0.04	Crush pressure (MPa)	125
Damage parameter D2	1.0	Compaction pressure (GPa)	6
Hugoniot polynomial coefficient A1 (GPa)	35.27	Shear modulus reduction factor	0.5
Hugoniot polynomial coefficient A2 (GPa)	39.58	Eroding plastic strain	2.0
Hugoniot polynomial coefficient A3 (GPa)	9.04	Minimum damaged residual	0.01
Failure surface parameter A	1.60	Porosity exponent	3.0
Failure surface parameter N	0.61	Initial porosity	1.0
Residual surface parameter AF	1.60	Pressure influence on plastic flow in tension	0.001
Residual surface parameter NF	0.61	Tensile strain rate dependence exponent	0.036
Parameter for polynomial EOS B0	1.22	Compressive strength (MPa)	167.8
Parameter for polynomial EOS B1	1.22	Compressive strain rate dependence exponent	0.032
Reference compressive strain rate	3E-05	Gruneisen gamma	0
Reference tensile strain rate	3E-06		

3 Simulation results and analysis

3.1 The influence of temporal thickness on crack propagation under geostress is not considered

Figure 2 illustrates the comprehensive development of peripheral cracks with different joint widths when subjected to explosive stress waves and explosive gas pressure, without taking into consideration the effects of in-situ stress ($\boldsymbol{\sigma}_{\mathrm{v}}$=$\boldsymbol{\sigma}_{\mathrm{h}}$=0). As depicted in Fig. 2(a), in the absence of any joints, an initial stage of the explosion leads to the formation of a small crushing zone near the two gun holes. Beyond this crushing zone, a radial crack zone emerges with random cracking patterns. The propagation of stress waves to the depth of the gun holes causes the gradual expansion of radial cracks, resulting in the formation of several primary and secondary cracks (350us). The crushing

zone arises due to the radial compressive stress exceeding the compressive strength of the rock, while the radial cracks stem from the circumferential tensile stress surpassing the tensile strength of the rock. As the stress wave travels and its energy gradually dissipates during the propagation process, the cracking effect weakens as well. At 350us, the stress wave intersects with the two gun holes. At this point, the crack propagation speed is slower than the stress wave propagation speed, and the crack between the gun holes has not fully penetrated. Subsequently, under the influence of explosive gas, the radial primary crack is further extended (550us, 750us), eventually resulting in the connection of the explosive crack between the two holes (1000us). This numerical simulation effectively recreates the entire process of the formation of a crushing zone, a fissure zone, and the interpenetration of cracks between the gun holes within the surrounding rock mass when subjected to blasting.

As depicted in Fig. 2(b~d), when a vertical open joint is present between the two gun holes, and the joint distance is equivalent to that between the two gun holes, a similar scenario to Fig. 2(a) unfolds under the influence of explosive stress waves, 350us earlier. In this case, the presence of the joint has a relatively minor impact on crack propagation. At 350us, in the absence of any joint, the stress wave meets and superimposes, elevating the stress at the center of the connection between the two holes. However, no rock precracking is observed at the center. With the presence of joints, it becomes challenging for stress waves to traverse through the joints. The existence of open joints leads to the reflection of compressive stress waves into tensile stress waves, resulting in the formation of a tensile failure zone at the joint plane. Rock near the joint plane experiences damage from both compressive and tensile stress waves. Cracks initiate at the center of the joints and compress the joints, causing the two joint surfaces to meet and then subjecting them to tensile forces. This process involves partial intersection and separation of the joint surfaces. From 350us to 1000us, the cracks continued to expand under the influence of explosive stress waves and explosive gas. After 550us, the rate of crack expansion decreased. At this point, the explosive stress wave had propagated to the end of the joint, and the distance between the joints shortened until the joint surfaces came into contact with each other.

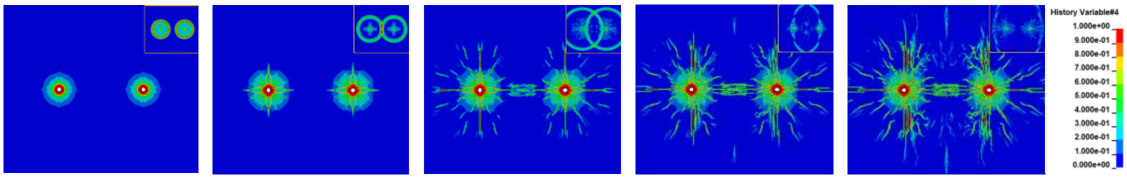

250us 350us 550us 750us 1000us

(a) Without joint

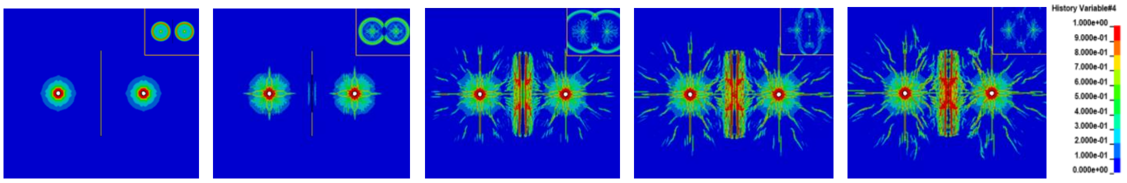

250us 350us 550us 750us 1000us

(b) joint thickness of 2cm

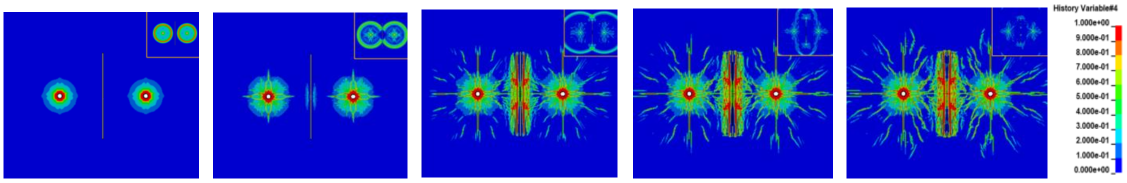

250us 350us 550us 750us 1000us

(c) joint thickness of 4cm

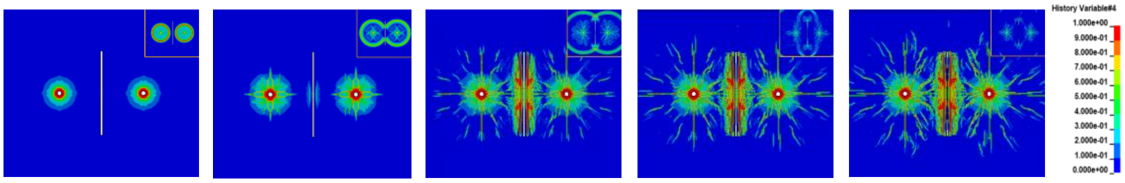

250us 350us 550us 750us 1000us

(d) joint thickness of 6cm

Fig 2 Evolution of explosive cracks in rock mass with different joint thicknesses without in-situ stress (stress wave cloud image at upper right corner)

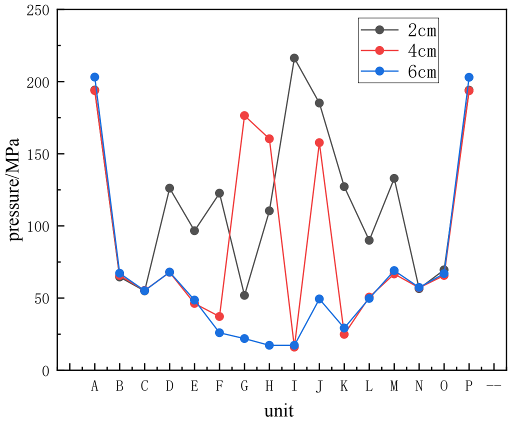

Fig 3 Peak stress curve of each unit

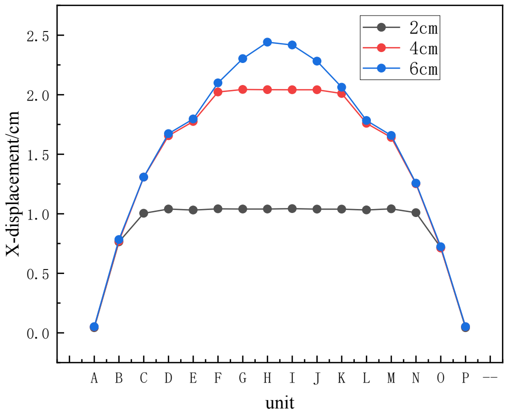

Fig 4 Peak displacement curve of each unit

As illustrated in Figure 3, stress peaks are extracted based on the selected elements. The stress variation pattern observed in the figure is approximately "W"-shaped. The stress profiles at the two ends of the joint almost mirror each other and align with the other two curves. When the joint thickness is 2cm, the maximum stress peak at the center is 216.2MPa, while the minimum stress is 51.9MPa. With a joint thickness of 4cm, the maximum stress at both ends of the joint is 193.7MPa, and the minimum stress is 16MPa. For a joint thickness of 6cm, the maximum stress at both ends of the joint is 203.1MPa, and the minimum stress is 17.3MPa. It's worth noting that the stress at both ends of the joint exceeds the compressive strength of the rock, resulting in rock failure, and the rock between the two ends predominantly experiences tensile stress.

In Figure 4, it's evident that the displacements at both ends of the joint remain relatively constant, irrespective of the joint thickness, and exhibit a symmetrical relationship. When the joint thickness is 2cm and 4cm, the displacement of the element converges within a horizontal range. This occurs because the small joint thickness leaves no space for movement between the joint surfaces upon contact. The maximum displacement is 1.04cm and 2.04cm, respectively. Conversely, when the joint thickness is 6cm, there's no contact between the two joint surfaces, and the maximum displacement is 2.44cm.

3.2 The effect of geological stress on crack propagation is not considered

As depicted in Figure 5(a), when there is a horizontal joint between the two holes, and the joint is not connected to the two holes, crack propagation at 250us under the influence of the explosive stress wave resembles the 250us scenario in Figure 1 and Figure 5(c~d). It results in the formation of a confined crushed zone and a radial micro-crack zone. The crack propagation tends to incline towards the end of the joint. With the passage of time, the existing radial micro-cracks continue to

spread further in their original direction. At 550us, horizontal cracks connect with the joint. As the cracks expand, both main and secondary cracks gradually form. The cracks near both sides of the joint plane continue to widen under the influence of the explosive gas, eventually breaking through. The number of cracks between the two holes exceeds those in the opposite direction and surrounds both sides of the joint plane. It's evident in the stress wave cloud diagram that, as the stress wave propagates to the joint's end, local damage occurs at the joint end. The presence of the joint alters the stress wave's propagation path. The stress wave propagates opposite to the joint plane, and stress attenuation near the joint plane accelerates with increasing distance. However, the rock mass on both sides of the joint plane remains largely unaffected. After the explosion crack connects with the horizontal joint, it significantly influences the subsequent crack propagation.

In Figure 5(b), when there is a joint inclined at 30° between the two gun holes, it substantially alters the rock's damage pattern. During the explosion stress wave stage, both ends of the joints near the gun holes experience the effect of the explosion stress wave. This causes the joints to move toward the back joint plane, leading to the creation of an area with higher crack density at the joint ends, which gradually extends away from the gun holes (250us). This behavior is due to the reflection of the stress wave when it reaches the joint surface, resulting in reflected tensile stress. As the stress wave reaches the joint end, it diffracts and generates a wing crack (350us) at the end. By 550us, the crack continues to expand along the initial crack, the wing crack keeps developing, and the crack at the joint end extends to the far end of the gun hole, connecting with the wing crack. Notably, there are almost no cracks near the hole. This is because the presence of the joint hinders the propagation of stress waves, and initial crack formation is impeded. As the stress wave propagates, its gradual attenuation becomes insufficient to cause rock damage (750us).

As illustrated in Figure 5(c), when there is a joint with a 60° inclination angle between the two gun holes, the side of the joint plane near the gun holes initially experiences the effects of compressive stress waves. It forms reflected tensile waves at 350us during the explosion stress wave stage. This stage leads to the formation of a crushed zone and a radial micro-crack zone around the gun holes and generates the main crack. However, there is no local damage occurring at the joint end during this phase. As the stress wave propagates to a greater distance, it gradually weakens. During this time, the explosive gas further extends the original crack on the side away from the joint plane, resulting in a shorter wing crack (550us). Simultaneously, the crack on the side near the joint plane hinders the expansion of the original main crack in that direction due to the barrier effect of the joint plane. The cracks extend along the direction of the joint surface (750us), leading to the formation of many secondary cracks and resulting in significant damage on both sides of the joint (1000us). Evidently, when the joint angle is 60°, it has a pronounced promoting effect on crack expansion, resulting in an effective blasting outcome.

In Figure 5(d), when there is a joint with a 90° angle between the two gun holes, the joint initially has no impact on crack propagation during the early stage of the explosion stress wave. As the stress wave reaches the joint surface at 350us, rock damage first occurs on both sides of the joint midpoint, forming a crushed zone around the gun holes and radial main cracks. When the stress wave propagates to the joint end, the existing main crack continues to propagate further in its original direction, and reflected tensile stress in the middle of the joint plane causes rock failure (550us). Between 750us and 1000us, there is no significant difference in crack propagation. However, the joint end guides the crack propagation within its region, and the explosive crack tends to incline toward the joint end during its expansion along the original direction. When comparing

Figure 2(a) and Figure 5(d), it becomes clear that when the angle between the joint and the line connecting the gun holes is 90°, the joint does not impede the crack penetration between the gun holes. Instead, it has a promoting effect on crack expansion, leading to a more even distribution of cracks on the joint surface and improved rock fragmentation. Further comparison of the evolution of explosive cracks at different joint angles reveals that the blasting effect is worst at a 30° angle. The test results of smooth blasting conducted by Wu Li et al[31] also indicate that the blasting effect is poorest when the angle between the rock mass's structural plane and the blasting fracture plane is approximately 30°. The simulation results in this paper align with these test findings.

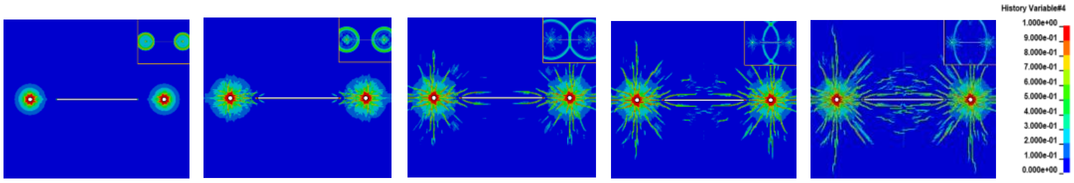

250us 350us 550us 750us 1000us

(a)Joint angle of 0°

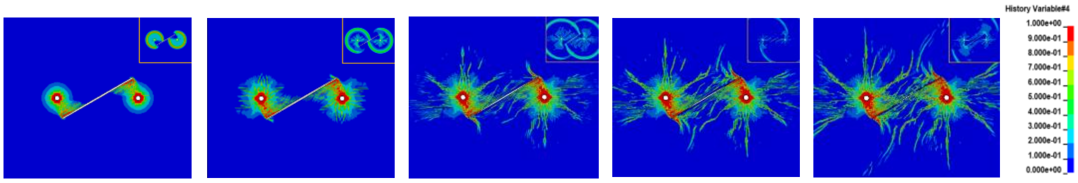

250us 350us 550us 750us 1000us

(b)Joint angle of 30°

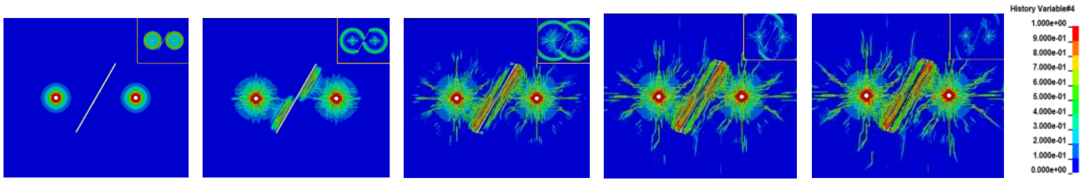

250us 350us 550us 750us 1000us

(c)Joint angle of 60°

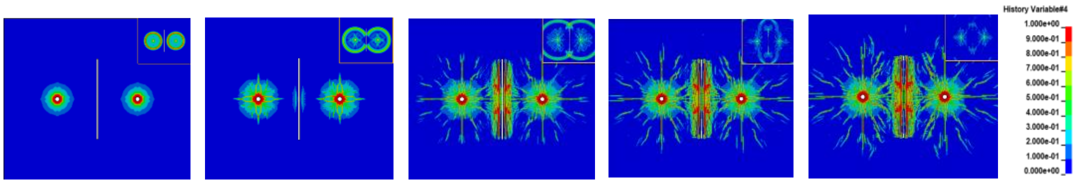

250us 350us 550us 750us 1000us

(d)Joint angle of 90°

Fig 5 Evolution of explosive cracks in jointed rock mass without in-situ stress

(stress wave cloud image at upper right corner)

3.3 Analysis of blasting effect of jointed rock mass under different in-situ stress conditions

Figure 6 illustrates the final distribution pattern of explosive crack growth under varying in-situ stress conditions. The key observations from this figure include: When there is no joint between two gun holes (depicted in Figure 6(a)), the growth of explosive cracks is primarily influenced by the in-situ stress condition. Notably, the distribution and length of cracks formed at different lateral

stress coefficients (λ = 0.5, 1, and 2) are smaller than those without in-situ stress. It's worth highlighting that the vertical crack length is more pronounced when λ = 0.5 than under other conditions. The horizontal cracks are suppressed, and vertical crack propagation prevails. Additionally, the cracks tend to incline between the two holes. In cases where λ = 1, both horizontal and vertical cracks experience inhibition. The number of secondary cracks is reduced, and the primary cracks are shorter. In contrast to scenarios with λ = 0.5, when λ = 2, there is a notable promotion of crack formation and propagation in the horizontal direction. More cracks are observed between the holes, and the suppression of primary crack growth in the vertical direction is intensified. In fact, there are virtually no secondary cracks in these conditions. These observations are primarily attributed to radial crack propagation resulting from circumferential tensile stress. When λ takes on values of 0.5, 1, and 2, the in-situ stress in all directions surrounding the rock mass is compressive stress, which tends to impede the initiation and propagation of cracks. These findings align with the results presented in the work of Dai Jun and Qian Qihu [32], which focused on parameters related to roadway caving and blasting under high in-situ stress conditions.

In the scenario where the joint angle is 0° (refer to Figure 6(b)), and in the absence of in-situ stress, we observe the following phenomena: Explosive cracks propagate in all directions, resulting in a higher number of secondary cracks. The primary cracks in the vertical direction shift towards the back of the joint due to the influence of stress waves. At a lateral stress coefficient of λ=0.5, the vertical cracks extend to the model's boundary, essentially running through the entire model. Horizontal cracks gather on both sides of the joint, with secondary cracks being relatively scarce and primarily concentrated between the two holes. For scenarios with λ=1 and λ=2, the primary cracks in the vertical direction tend to propagate towards the joint, driven by the effects of in-situ stress. This behavior substantially inhibits the initiation of secondary cracks. The inhibitory effect is more pronounced with λ=2, where in-situ stress plays a stronger role in suppressing crack growth. As a result, the initiation and propagation of secondary cracks are significantly hindered under conditions of in-situ stress. Now, when the joint angle is 30° (as shown in Figure 6(c)), the explosive crack extends towards the joint's end, forming a wing crack on the back joint plane during the propagation process. This crack extends away from the gun hole on the side closest to the gun hole and intersects with the wing crack generated by another gun hole. While the initial in-situ stress still has some inhibitory effect on secondary crack initiation and propagation, this effect is less pronounced than for other joint angles. Notably, there are no cracks observed at both ends of the joint, which is attributed to stress concentration within the joint. In summary, the presence of initial in-situ stress and a joint angle of 30° results in the joint playing a prominent role in guiding and promoting explosive crack propagation. Under these conditions, the joint primarily controls the direction of crack propagation.

In the case where the joint angle is 60° (as depicted in Figure 6(d)), we observe the following behaviors: Secondary cracks are inhibited, and the primary crack expands towards the direction of the maximum principal stress. The rock damage on the joint plane closest to the gun hole is more extensive, and the cracks are predominantly oriented towards both ends of the joint. Under conditions of lateral stress coefficients λ=0.5 and λ=1, a long primary crack originates from the joint. These cracks almost traverse the entire model, although the suppression of primary cracks at the hole varies. With λ=2, it is evident that the inhibitory effect on crack propagation in the vertical direction is more pronounced, favoring horizontal crack growth. When the joint angle is 90° (as shown in Figure 6(d)), for lateral stress coefficients λ=0.5 and λ=1, crack propagation on both sides

of the joint tends to incline towards the direction of the gun hole. However, with λ=2, the cracks extend towards the joint's end, and the joint exhibits a clear guiding effect on the explosive cracks. This phenomenon results from the combined influence of joint stress and joint guidance, with the direction of crack propagation being influenced by both the joint and the initial in-situ stress. To summarize, the specific joint angle and lateral stress coefficients significantly affect the patterns of crack propagation, with different conditions leading to variations in crack distribution, orientation, and the extent of primary and secondary cracks in the rock mass. These observations highlight the complex interplay of geological and stress factors in controlling the blasting process.

	Without in-situ stress	□=0.5	□=1	□=2
(a)without joint	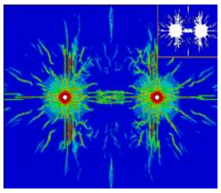	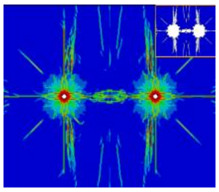	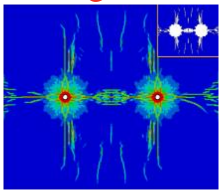	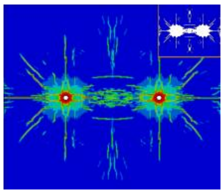
(b)0°	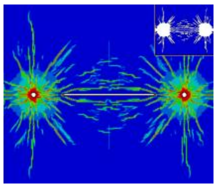	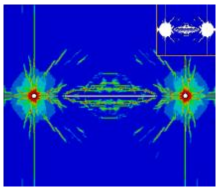	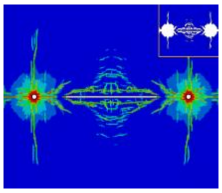	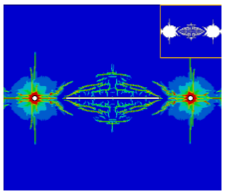
(c)30°	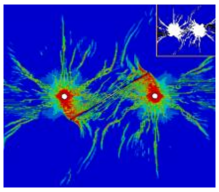	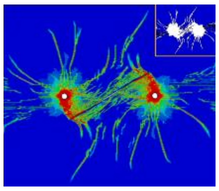	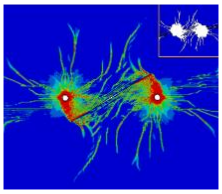	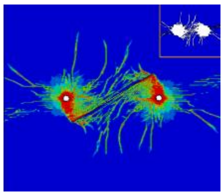
(d)60°	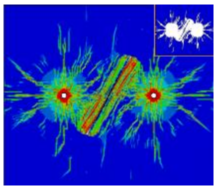	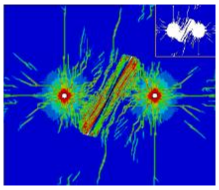	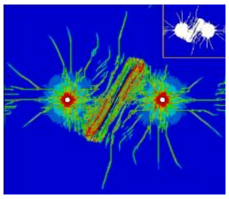	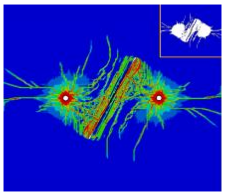
(e)90°	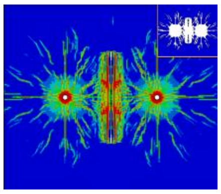	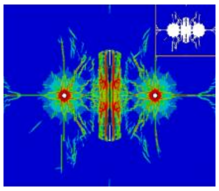	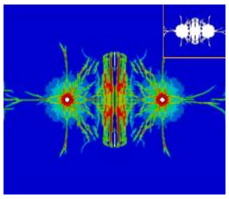	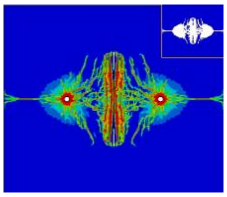

Fig 6 Distribution diagram of crack growth in rock mass under different in-situ stress conditions

(damage range diagram in the upper right corner)

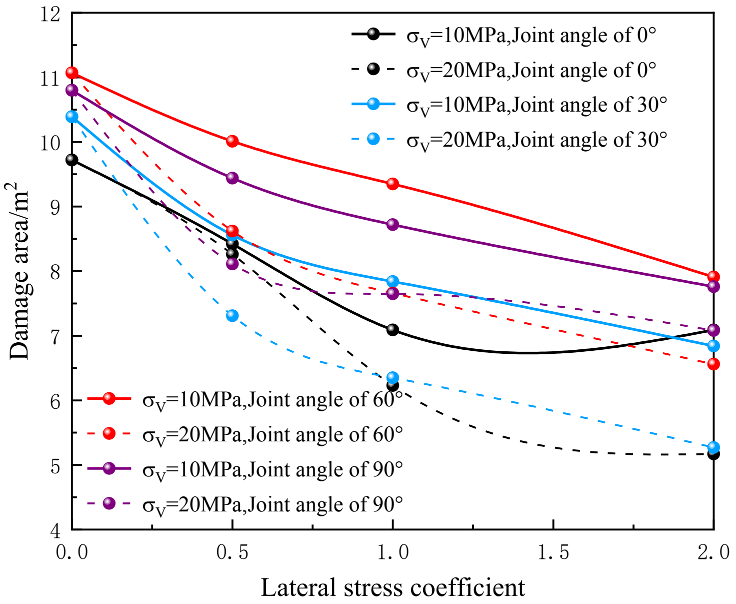

Fig 7 The area of blasting damage zone formed under different conditions

Figure 7 presents the changes in the area of the blasting damage zone under different in-situ stress conditions and joint angles. When the lateral stress coefficient λ remains constant and the burial depth is increased, variations in blasting effectiveness are observed. To facilitate meaningful comparisons, this study incorporates diverse in-situ stress values for a consistent lateral stress coefficient. It also considers the absence of a joint as the baseline for the damage area when the joint angle is 0°, although it should be noted that this specific scenario deviates from other blasting models in terms of hole positions. As depicted in Figure 6, it becomes evident that maintaining a fixed lateral stress coefficient λ while elevating the vertical in-situ stress from 10MPa to 20MPa results in decreased crack expansion length and a reduced damage area. Clearly, as the burial depth increases and the in-situ stress intensifies, the initiation and expansion of cracks become more restricted. This conclusion aligns with the findings reported by Yang Jianhua and their colleagues [33]. In summary, the interaction of varying in-situ stress and joint angles has a pronounced impact on the extent of damage caused by blasting. The study reveals the complex relationship between these factors and provides valuable insights into optimizing blasting procedures in different geological conditions.

Figure 7 presents the area of the blasting damage zone under various conditions, taking into account vertical in-situ stress $\boldsymbol{\sigma}_{\mathrm{v}}$ at 10MPa and 20MPa, lateral stress coefficients at 0.5, 1, and 2, and joint angles at 30°, 60°, and 90°. Notably, when holding the lateral stress coefficient constant and varying the joint angles, the damage zone area tends to increase with an increasing joint angle. However, it's crucial to recognize that the damage zone area doesn't exhibit indefinite growth with larger angles; instead, there appears to be an optimal value. In this study, the most extensive blasting damage area within the rock mass was observed when the joint angle was set at 60° and $\boldsymbol{\sigma}_{\mathrm{v}}$ was 10MPa. Additionally, jointed rock masses display larger damage areas compared to unjointed ones, and the presence of a joint results in the smallest damage area when the joint angle is 30°. In conclusion, the research sheds light on the interplay between in-situ stress conditions, joint angles, and blasting damage. These findings provide valuable insights into the optimization of blasting practices across various geological contexts.

4 Conclusion

In this study, numerical simulations of the blasting process in jointed rock masses under

various conditions were conducted, yielding the following main conclusions:

(1) When an open joint is present in the rock mass, and the joint thickness doesn't align with the joint plane, maximum element displacement occurs at the joint plane. Stress concentration is observed at both ends of the joint, while the stress between the two ends is relatively mild, resulting in a larger damage area. When the joint thickness is too small, maximum stress occurs at the center of the hole connection. The presence of a joint exerts a significant guiding effect on crack propagation.

(2) The existence of a joint imposes a barrier to crack expansion and penetration. When the explosion stress wave reaches the joint surface, it hinders the propagation of the stress wave, leading to the diffusion of wing cracks at the joint end. Under similar lateral stress coefficients and joint angles, the area of the blasting damage zone decreases with increasing initial in-situ stress.

(3) Under the same initial in-situ stress conditions, the area of the blasting damage zone initially increases and then decreases as the joint angle increases. However, it remains larger than that in the absence of a joint, and there is an optimal angle that maximizes the damage area. In simulated conditions, the largest damaged crack area is observed when the joint angle is 60°.

(4) The presence of initial in-situ stress has a certain impact on the initiation and expansion of blasting cracks. The degree and pattern of this influence are not only related to the lateral stress coefficient but also associated with the joint's angle and thickness. When in-situ stress is present, the pressure effect from the initial in-situ stress field doesn't favor the initiation and propagation of blasting cracks. Nevertheless, the presence of joints exhibits a clear guiding and promoting effect on crack propagation. The morphology of crack propagation is jointly controlled by both the joints and in-situ stress conditions.

Acknowledgments

This work was supported by the National Natural Science Foundation of China Project No. 51904145, Basic scientific research project (youth project) of Liaoning Provincial Department of Education in 2022 No. LJKQZ20222322, the Engineering Laboratory of Deep Mine Rockburst Disaster Assessment Open Project No. LMYK2020006, the Liaoning Natural Science Foundation Program Guidance Plan No. 2019-ZD-0045, and the Liaoning Provincial Department of Education Project No. LJ2019JL007.

References:

[1] Wu Bo. Study on disturbance zone evolution and anchoring mechanism of surrounding rock in layered rock tunnel[D]. Wuhan: China University of Geosciences, 2016: 1-2.

[2] LISJAK A, GRASSELLI G, VIETOR T. Continuum discontinuum analysis of failure mechanisms around unsupported circular excavations in anisotropic clay shales[J]. International Journal of Rock Mechanics and Mining Sciences, 2014, 65(19): 96-115.

[3] Han Zhiming, Qiao Chunsheng, Xu Hongliang. Analysis of strength anisotropy of rock mass with a set of persistent joints[J]. Journal of China University of Mining & Technology, 2017, 46(5): 1073-1083.

[4] Yang Zhongmin, Gao Yongtao, Wu Shunchuan, et al. Study of the influence of joint parameters on rock mass strength based on equivalent rock mass technology[J]. Journal of China University of Mining & Technology, 2018, 47(5): 979-986.

[5] Cheng Jianlong, Yang Shengqi, Yin Pengfei, et al. Experimental study of the deformation and strength behavior of composite rock specimens in unloading confining pressure test [J]. Journal of China University of Mining & Technology, 2018, 47(6): 1233-1242.

[6] Dai Jun. Dynamic characteristics of rock and blasting theory[M]. Beijing: Metallurgical Industry Press, 2002.

[7] Xia Wenjun, Lu Wenbo, Chen Ming, et al. Study on safety threshold of peak particle velocity about blasting damage of columnar jointed basalt rock mass in Baihetan Dam site. Chinese Journal of Rock Mechanics and Engineering, 2019, 38(S1): 2997- 3007 (in Chinese).

[8] Varma M，Maji VB，Boominathan A. Influence of rock joints on longitudinal wave velocity using experimental and numerical techniques. International Journal of Rock Mechanics and Mining Sciences, 2021, 141: 104699.

[9] Wang Z L, Konietzky H. Modelling of blast-induced fractures in jointed rock masses. Engineering Fracture Mechanics, 2009, 76(12): 1945-1955.

[10] Yang P, Lei Q H, Xiang J H, et al. Numerical simulation of blasting in confined fractured rocks using an immersed-body fluid-solid interaction model. Tunnelling and Underground Space Technology, 2020, 98: 103352.

[11] Wang Z L, Wang H C, Wang J G, et al. Finite element analyses of constitutive models performance in the simulation of blast-induced rock cracks. Computers and Geotechnics, 2021, 135: 104172.

[12] Zhu J B, Zhao X B, Li J C, et al. Normally incident wave propagation across a joint set with the virtual wave. Journal of Applied Geophysics, 2011, 73(3): 283-288.

[13] Do TN, Wu JH. Verifying discontinuous deformation analysis simulations of the jointed rock mass behavior of shallow twin mountain tunnels. International Journal of Rock Mechanics and Mining Sciences, 2020, 130: 104322.

[14] Chai Shaobo, Li Jianchun, Zhao Junhai, et al. Study on stress P-wave propagation across intersecting rock joints with nonlinear deformation. Chinese Journal of Rock Mechanics and Engineering, 2019, 38(6): 1149-1157.

[15] Wang Shumin, Wang Zhiliang, Jia Shuailong, et al. Analysis of propagation characteristics of stress waves in viscoelastic jointed rock mass. Journal of Harbin Institute of Technology, 2022, 54(2): 99-107 (in Chinese).

[16] Xu Bangshu, Zhang Wangzhi, Shi Weihang, et al. Experimental study of parameters of tunneling blasting in jointed layered rock mass[J]. Journal of China University of Mining & Technology, 2019, 48(06): 1248-1255.

[17] Niktabar S M M, Rao K S, Shrivastava A K. Effect of rock joint roughness on its cyclic shear behavior[J]. Journal of Rock Mechanics and Geotechnical Engineering, 2017, 9(6): 1071-1084.

[18] Roy D G, Singh T N, Kodikara J. Influence of joint anisotropy on the fracturing behavior of a sedimentary rock[J]. Engineering Geology, 2017, 228: 224-237.

[19] Yang R S, Ding C X, Yang L Y, et al. Model experiment on dynamic behavior of jointed rock mass under blasting at high-stress conditions[J]. Tunnelling and Underground Space Technology, 2018, 74: 145-152.

[20] MIRANDA L, CANTINI L, GUEDES J, et al. Applications of sonic tests to masonry elements: influence of joints on the propagation velocity of elastic waves[J]. Journal of Materials in Civil Engineering, 2013, 25(6): 667-682.

[21] Singh D P, Sastry V R. Role of weakness planes in bench blasting-a critical study / / Process of the 2nd International Symposium on Rock Fragment by Blasting. Keystone,1987.

[22] Xie Bing, Li Haibo, Wang Changbai, et al. Numerical simulation of presplit blasting influenced by geometrical characteristics of joints[J]. Rock and Soil Mechanics, 2011, 32(12): 3812-3820.

[23] Qu Shijie, Liu Jifei. Numerical analysis of joint angle effect on cracking with presplit blasting[J]. Rock and Soil Mechanics, 2015, 36(1): 189-204.

[24] Wei Chenhui, Zhu Wancheng, Bai Yu, et al. Numerical simulation of jointed rock mass blasting under different in-situ stress conditions[J]. Chinese Journal of Engineering, 2016, 38(1): 19-25.

[25] Zhang Fengpeng, Peng Jianyu, Fan Guanghua, et al. Mechanisms of blasting-induced rock fractures under different static stress and joint properties conditions[J]. Rock and Soil Mechanics, 2016, 37(7): 1839-1846，1913.

[26] Deng X F, Zhu J B, Chen S G, et al. Numerical study on tunnel damage subject to blast-induced shock wave in jointed rock masses[J]. Tunnelling and Underground Space Technology, 2014, 43(6): 88-100.

[27] Shao Peng, Dong Zhaoxing，Zhang Yong. Comment on the research of rock blast models[J]. Rock and Soil Mechanics, 1999(03): 91-96.

[28] Li, X, Zhu Z, Wang M, Wan D, Zhou L & Liu R (2021). Numerical study on the behavior of blasting in deep rock masses. Tunnelling and Underground Space Technology, 113, 103968.

[29] Wang, Z, Wang H, Wang J & Tian N. (2021). Finite element analyses of constitutive models performance in the simulation of blast-induced rock cracks. Computers and Geotechnics, 135, 104172.

[30] Li X, Zhu Z, Wang M, et al. Numerical study on the behavior of blasting in deep rock masses[J]. Tunnelling and Underground Space Technology, 2021, 113: 103968.

[31] Wu Li, Tao Xian. Theoretical analysis and experimental study of the influence of rock structural planes on blasting effect. Mining Research and Development, 1999, 19(1): 40-43.

[32] Dai Jun, Qian Qihu. Break blasting parameters for driving a roadway in rock with high residual stress. Explosion and Shock Waves, 2007, 27(3): 272-277.

[33] Yang Jianhua, Lu Wenbo, Hu Yingguo, et al. Accumulated damage in surrounding of tunnels. Rock Soil Mech, 2014, 35(02): 511-518.

Figure Click here to access/download;Figure;Figure.docx

Cover Letter

1.The corresponding author's name, institutional affiliation, address, and telephone number.

Name: Hai Rong
Affiliation: College of Mining, Liaoning Technical University, China
Address: Liaoning Technical University, No. 47 Zhonghua Road, Fuxin City, Liaoning Province, People's Republic of China
Email: ronghai1988@163.com
Tel: +8615841818211

2.Co-author information

Name: Nannan Li
Affiliation: College of Mining, Liaoning Technical University, China
Address: Liaoning Technical University, No. 47 Zhonghua Road, Fuxin City, Liaoning Province, People's Republic of China
Email: 934744832@qq.com

Name: Chen Cao
Affiliation: College of Mining, Liaoning Technical University, China
Address: Liaoning Technical University, No. 47 Zhonghua Road, Fuxin City, Liaoning Province, People's Republic of China
Email: 2065214306@qq.com

Name: Yadi Wang
Affiliation: College of Mining, Liaoning Technical University, China
Address: Liaoning Technical University, No. 47 Zhonghua Road, Fuxin City, Liaoning Province, People's Republic of China
Email: 326040139@qq.com

Name: Jincheng Li
Affiliation: College of Mining, Liaoning Technical University, China
Address: Liaoning Technical University, No. 47 Zhonghua Road, Fuxin City, Liaoning Province, People's Republic of China
Email: 1404600267@qq.com

Name: Mingda Li
Affiliation: College of Mining, Liaoning Technical University, China
Address: Liaoning Technical University, No. 47 Zhonghua Road, Fuxin City, Liaoning Province, People's Republic of China
Email: 1269631235@qq.com

3. The names and contact details of any reviewers I consider suitable.

First name/Given name: Cun
Surname/Family name: Zhang
School of Energy and Mining Engineering
China University of Mining and Technology, Beijing
Xueyuan Road D11, Beijing 100083, PR China
Tel: +8615810194127
Email: cumt-zc@cumtb.edu.cn

First name/Given name: Kunyou
Surname/Family name: Zhou
School of Mining Engineering, Anhui University of Science & Technology
Tel: +8617851983158
Email: TB18020033B4@cumt.edu.cn

First name/Given name: Shengquan
Surname/Family name: He
School of Civil and Resource Engineering, University of Science and Technology Beijing
Tel: +8617801187251
Email: shenqhe@163.com

First name/Given name: Mengda
Surname/Family name: Wu
School of Mining Engineering, Taiyuan University of Technology
Email: wuwenda@tyut.edu.cn
