# Supplementary material for: Numerical simulation of rock blasting under different in-situ stresses and joint conditions
Source: PLoS One. 2024 Apr 22;19(4):e0299258. doi: 10.1371/journal.pone.0299258 (PMC11034639; doi:10.1371/journal.pone.0299258)
Supplement: Attachment — Submitted filename: Response to Reviewers.docx [file pone.0299258.s002.docx]

**Reply to the first reviewer's comments**

Dear reviewer,

Thank you very much for reviewing our paper, and providing comments and suggestions to us. I am Nannan Li, the corresponding author of the paper “Numerical simulation of rock blasting under different in-situ stresses and joint conditions (PONE-D-23-39104)”

I have revised my manuscript according to the changes requested by the reviewer, and now I will reply to the comments item by item. Thank you！

**Comment 1:**

**The summary lacks emphasis on aspects of novelty.**

Answer 1:

Thank the reviewer for his valuable comments.

You correctly point out the lack of emphasis on novelty in the abstract. The following is a revised partial abstract, highlighting the novelty of the study:

“... Numerical simulation model. In this study, the effects of lateral stress coefficient, joint width, joint Angle and other parameters on crack generation and propagation of rock mass are studied by finite element numerical simulation. Through stress analysis and damage area comparison, the relationship between damage crack growth and horizontal and vertical stress difference of rock mass is explored, and the action principle of these factors is analyzed. ...”

Thanks to the reviewers.

**Comment 2:**

**The introduction chapter should be numbered 1, not 0.**

Answer 2:

Thank the reviewer for his valuable comments.

The author has revised the Revised Manuscript with Track Changes according to your comments.

Thanks to the reviewers.

**Comment 3:**

**The literature review is not complete, a number of works aimed at the study of rock properties, the choice of constitutive models, or how to describe the process of interaction of, for example, the blast wave with the rock mass are missing, I encourage you to study some of the following works: DOI:10.1016/j.jrmge.2021.12.022,DOI:10.1016/j.ijimpeng.2022.104484,**

**DOI:10.1016/j.ijimpeng.2020.103543,**

**DOI:10.1016/j.jrmge.2020.09.007orDOI:10.1016/j.ijmecsci.2022.107197.**

Answer 3:

Thank you very much for your comments and recommendations of relevant literature.

Thanks to the reviewers for the relevant research papers. By studying these literatures, I have a more comprehensive understanding of the study of rock properties, the selection of constitutive models and the interaction between explosion waves and rock mass. These literatures provide important reference and guidance for my research. I have quoted these documents in the revised draft to better supplement the completeness of the literature review in the manuscript, and marked them in blue font.

Thanks to the reviewers.

**Comment 4:**

No consideration of residual stress state. I encourage you to study the paper: DOI:10.1155/2019/2878969.

Answer 4:

Thank the reviewer for his valuable comments.

You mentioned that the effect of the residual stress state was not considered, and I have read the paper you recommended and learned more about the research in this area. In my research, the state of residual stress is not the focus of the research, but the influence of ground stress on the development and propagation of cracks in jointed rock mass is mainly considered.

Through reading this paper, I realized the influence of the state of residual stress on the behavior of rock mass. In order to improve and deepen my research, I plan to explore more deeply the influence of residual stress on the materials or systems under study in future work. I will also consider more comprehensively the effects of various stress states on the behavior of materials in order to provide a more accurate and complete analysis. At the same time, I will quote the paper you recommended in my research to fully reflect the influence of the residual stress state on the research and ensure that my research results are more complete and accurate.

Thank you very much for the reviewer's guidance and the recommendation of relevant literature.

**Comment 5:**

There is no conversion of the process of interaction of the blast wave with the rock (section) and the effects associated with it - multiple reflections of the wave in the hole and overlapping of pressure waves or vacuum effect.

Answer 5:

Thank you very much for your comments.

In my research, I mainly study the interaction process between explosion shock wave and rock from a macroscopic perspective. In the research process, the interaction is mainly reflected in crack initiation, expansion and stress wave dissipation of rock mass. Secondly, the influences of the width, Angle of joint and lateral stress coefficient on crack propagation of rock mass are discussed.

Your guidance is very important, including the multiple reflections of shock waves in rock pores and the complexity of the propagation path, which is not viewed from a microscopic perspective in my research content. In the future research, I will further explore the physical process of shock wave propagation and rock interaction, including the multiple reflections of waves in holes and the propagation path of waves. This will contribute to a more comprehensive understanding of the impact of shock waves on rocks and improve the scientific research.

Thanks to the reviewers.

**Comment 6:**

A key aspect is missing, namely, an examination of the sensitivity of the model to initial-boundary conditions and the density of the FEM mesh. A key parameter in describing the destruction/cracking process of rock material.

Answer 6:

First of all, thank you very much for your comments. You correctly pointed out that my research lacks the sensitivity of initial boundary conditions and the sensitivity of finite element mesh density. And, you also mentioned that I need to describe the key parameters of the rock material failure/cracking process in the study. Here are the responses to your suggestions:

First of all, in this study, I set the initial boundary conditions, set the boundary of the model as non-reflective boundary conditions, simulate the infinite domain of the rock mass, and set the normal constraint in the Z direction. Secondly, the grid Quality Check is to evaluate the grid quality through the LS-PrePost internal function, click EleTol - EleEdit, and then select Check - Quality Check. There are many indicators to evaluate the quality of the grid, including Aspect Ration, Jacobian, Warapage and so on. The following is a model as an example, solid unit volume inspection, free solid unit inspection, repeated solid unit inspection, etc. Then, in the simulation process, the dynamic tensile strength, dynamic compressive strength and plastic strain of rock are mainly used as the key parameters of rock material failure/cracking.

In future studies, I will conduct more sensitivity analyses to assess the model's response to initial boundary conditions. I will study the effect of these conditions on the failure and cracking process of rock mass by varying different combinations of the initial boundary conditions, such as the initial stress and strain conditions. This will help me understand changes in the behavior of rock materials and the stability of models.

Thanks to the reviewers.

**Comment 7:**

There is no consideration of the heterogeneity of the rock material by taking into account cracks, defects or inclusions.

Answer 7:

Thank you for your valuable comments. Your comments are very valuable.

First of all, the heterogeneity of rock materials has a certain impact on crack propagation and the magnitude of ground stress. However, this study mainly takes homogeneous rock mass as the research object. In local rock mass, it is considered to be isotropic and has a kind of rock property, such as granite. In future studies, I plan to introduce more data and models on rock heterogeneity to more fully analyze the effect of ground stress on crack propagation.

Secondly, the cracks, defects or inclusions you mentioned are another important factor, which can also have a significant effect on the fracture and crack growth of the rock. The existence of these factors may change the mode of action of earth stress. In this paper, I think that the open joint can be regarded as one of the cracks and defects in rock mass. However, the combination of cracks and defects is very different, and my consideration is not comprehensive. At present, our consideration focuses on starting from the homogeneous rock mass, thoroughly studying its composition and other aspects, and then using the control variable method to add cracks, defects or inclusions one by one to turn them into a combination of parameters. This is the content of my next research, so as to more accurately evaluate the influence of different parameter combinations on rock behavior.

Thanks to the reviewers.

**Comment 8:**

There is no reference to experimental testing - no description of the validation process.

Answer 8:

Thank you for your valuable comments. Your comments are very valuable.

You pointed out that I did not mention the validation process of experimental tests in the study, and I recognize that this is an area that needs to be added to enhance the reliability of the study. However, there are many factors to consider in the existing laboratory tests, such as the limitation of confining pressure applied by rock mass and the harm of explosion wave to the experimenter. In the future research work, I will actively consider conducting experimental tests and describing experimental equipment, methods and results in detail to verify the accuracy and reliability of numerical simulation results.

Thanks to the reviewers.

**Comment 9:**

There is no analysis in terms of the sequence of ignition initiation - the sequence of explosions delays and what effects this has.

Answer 9:

Thank the reviewer for his valuable comments.

First of all, the sequence of ignition has an important effect on the explosion effect and result. It is a limitation of the study to analyze the sequence of initiation points. In this study, initiation mode is not taken as the focus of this research. In order to simplify the model, a quasi-two-dimensional model is adopted in the research process, and the initiation point is not defined, and the whole initiation mode is adopted. In the following research, 3D modeling will be used, and the initiation point will be defined, different initiation sequences will be considered and their impact on the results will be analyzed. This will lead to a better understanding of the explosion sequence and the effect of explosion wave effects on rock behavior.

Secondly, explosion delay is also an important factor in the study. In the current study, I did not consider the timing and delay of the explosion. In future studies, I will further study the timing and delay of the explosion and analyze its impact on the results to provide a more comprehensive and accurate analysis of the blasting effect.

Thanks to the reviewers.

**Reply to the second reviewer's comments**

**Comment 1:**

It is recommended to make amendments to the abstract by providing more detailed information on the methodology while minimizing the emphasis on findings. The conclusions already provide a comprehensive overview of the results.

Answer 1:

Thank the reviewer for his valuable comments.

In the revised draft, I have revised it according to the teacher's comments and provided more detailed information about the method. The revised content has been marked in blue font.

Thanks to the reviewers.

**Comment 2:**

Improve Figure 1 (looks overlapping)

Answer 2:

Thank the reviewer for their valuable comments.

I have revised Figure 1 while revising the manuscript and track changes.

Thanks to the reviewers.

**Comment 3:**

The rock types used for simulation.

Answer 3:

Thank the reviewer for their valuable comments.

In 2.3, I modified the relevant part and marked it with blue font.

Thanks to the reviewers.

**Comment 4:**

Edit Figure 6 (Lambda)

Answer 4:

Thank the reviewer for their valuable comments.

I have revised Figure 6 while revising the manuscript and track changes.

Thanks to the reviewers.

Thank you for giving me the chance to revise our manuscript!

Best regards,

Nannan Li

Liaoning Technical University, China.
