# Supplementary material for: Numerical simulation of rock blasting under different in-situ stresses and joint conditions
Source: PLoS One. 2024 Apr 22;19(4):e0299258. doi: 10.1371/journal.pone.0299258 (PMC11034639; doi:10.1371/journal.pone.0299258)
Supplement: Attachment — Submitted filename: Response to Reviewers .docx [file pone.0299258.s003.docx]

Many thanks to the review teachers and the editorial department for providing valuable suggestions for revision. Here are the answers to the questions:

Reviewer 1：additional information on the numerical model

Thank the reviewer for his valuable comments.

Complete digital model information is provided in the manuscript.

Thanks to the reviewers.

Reviewer 2：the relevance of the cited references

DOI: 10.1016 / j.jmecsci.2022.107197, this paper is different from the constitutive model calculation method of the manuscript, but both are useful for the study of damage parameters and cracks, which is related to the manuscript.

DOI: 10.1016 / j.jimpeng.2020.103543, this paper introduced the determination method of the parameters of the dolomite JH-2 model, which is conducive to future research and reference.

DOI: 10.1016 / j.jimpeng.2022.104484, this paper evaluated the performance of the three models under different stress conditions, and the manuscript has some similarities with them, which is relevant and conducive to further research.

DOI: 10.1016 / j.jrmge.2021.12.022, the study on the effects of heterogeneity and initial cracks on failure and cracking modes in this paper is related to the content of the manuscript, and both have initial cracks, which is very valuable for reference research.

DOI:10.1155/2019/2878969, this paper is a multi-scale modeling of rock mass and lays the foundation for the initial conditions of the three-dimensional finite element model. The initial conditions of rock mass are also considered in the manuscript.

To sum up, I think the added references are related to the manuscript, so quoting the papers proposed by the reviewer is conducive to further research.

Thanks to the reviewers.

During self-examination, it was found that the original references 33 and 35 were repeated in the Manuscript, and the revisions were made in the reply Revised Manuscript with Track Changes 2.
